# The Denver Meeting

**Published:** 1898-07

**Authors:** 


### EDITORIAL.

The office of THE JOURNAL is 305 and 306 Fitten Building.

Address all communications, and make all remittances payable to THE ATLANTA MEDICAL AND SURGICAL JOURNAL, Atlanta, Ga.

Reprints of articles will be furnished, when desired, at a small cost. Requests for the same should always be made *on the manuscript.*

THE senior editor, Dr. Grandy, has been appointed by the Governor surgeon of the Third Georgia Regiment of Volunteers, with the rank of major. The duties of the place will take him away from Atlanta for an indefinite period, during which the editorial work of the JOURNAL will be performed by his friend and colleague, Dr. Dunbar Roy.

### THE DENVER MEETING.

The Denver meeting of the American Medical Association was in many respects one of the most enjoyable meetings ever held by that body. The profession and people of Denver did everything to make the meeting the grand success which it proved to be. The dinners, receptions and entertainments were of a very high order and unusually enjoyable, but there is a growing feeling that these entertainments should be curtailed, as they take a great deal of time that might be employed to much better advantage in the work of the Association. The suggestion made in the Journal of the American Medical Association that the general sessions be held at night, in lieu of the receptions, strikes us as a good one.

The American Medical Editors Association met on June 6th, and on the evening of June 6th the profession of Denver gave the

American Medical Editors Association and invited guests a very handsome dinner at the Brown Palace Hotel, which was most delightful in every respect. Dr. I. N. Love, of St. Louis, acted as toast-master in his usual graceful and winning style, and the responses to the toasts were very entertaining. The editors seemed in a jolly mood, and popular patriotic songs and airs were received by them with great enthusiasm.

The joint section dinner of the Surgical and Gynecological sections at the Brown Palace Hotel Tuesday evening was the most largely attended and delightful of the section dinners. Dr. W. L. Rodman, of Louisville, as toast-master did himself and the sections great credit by the very graceful manner in which he presided over the dinner.

The general sessions of the Association were very interesting indeed. The address on general medicine by Dr. Musser, of Philadelphia, was a most classical and entertaining paper.

The address on surgery by Dr. Murphy, of Chicago, excited unusual interest on account of its novelty, and will make an epoch in the treatment of pulmonary tuberculosis if his deductions are correct. He takes the position that the surgical principle of rest should be applied to the treatment of pulmonary tuberculosis just as to other local tuberculoses. Dr. Murphy seems to have demonstrated that the sudden deaths which follow opening the chest cavity are due to the vibration of the lung, and that by holding the root of the lung steady, with forceps, this issue can be prevented. He was led to suggest this plan of treatment by the fact that as 80 per cent. of the cases of pleurisy are tubercular, and as a very large proportion of the worst cases terminating in empyema are cured by the operation of removing sections of the ribs allowing the chest wall to collapse, consumption ought to be cured by bringing about a somewhat similar state of affairs artificially. He was led to the use of nitrogen gas because of its indefinite and non-irritating properties. He injects the pleural cavity with this gas, compressing the lung freely, and claims very great relief to cough and other distressing symptoms. The nitrogen gas may be removed after it has accomplished its mission by aspiration.

A sufficient number of cases have not been treated for a sufficient length of time to prove the correctness of Dr. Murphy's theory, or the advisability of adopting the plan of treatment suggested by him. So many cures for pulmonary tuberculosis have been discovered and published to the world, which have proven failures, that I am somewhat skeptical as to the value and practicability of the plan of treatment suggested by Dr. Murphy.

The side trips "around the loop," to Colorado Springs and Pikes Peak, arranged for the delegates were very delightful outings, and a large number availed themselves of these privileges.

The large number who attended the Denver meeting shows what great interest the profession at large feel in the American Medical Association to go in such numbers to such a distance to attend the meeting. It is a very great privilege to be a member of the American Medical Association, and while the Association is growing in strength and numbers every year, it should include in its membership every reputable physician in the United States. By becoming a permanent member one avails himself of all the privileges of the Association and gets one of the very best medical journals, if not the best, published in the United States, all for $5.00 per annum.

There is one thing that left a very bad impression on many of the members and delegates who attended the Denver meeting, and that was the disposition to gouge manifested in certain quarters. This was especially true of some of the hotels, and there was very general complaint of the inadequacy of the hotel facilities and of the outrageous charges made in many instances. I know nothing personally of the conduct of any of the hotel authorities except of the Albany. I have never received as poor entertainment anywhere for the money as I did at the Albany. The table service was miserable, the food very bad, and the tendency to get as much for as little as possible apparent at every turn. The hotel authorities absolutely disregarded positive contracts made with parties prior to the meeting. One gentleman who paid his bill at the same time that Dr. Murphy, of Atlanta, paid our bill, had a written contract with the hotel to furnish room and board for himself, wife and son, to which the clerk refused to pay any attention whatever, and charged him just as though no arrangement had been made.

This kind of conduct on the part of hotels where the meetings are held is simply outrageous, and it is sincerely hoped that the Committee of Arrangements at future places of meeting will see to it that such impositions are not again practiced. The meetings should not be held anywhere unless there is a guarantee of proper entertainment of the Association by the hotels at the usual rates.

The next meeting will be held in Columbus, Ohio, on the first Tuesday in June, 1899. F. W. M.

